# The Simplified Carbapenem Inactivation Method (sCIM) for Simple and Accurate Detection of Carbapenemase-Producing Gram-Negative Bacilli

**DOI:** 10.3389/fmicb.2018.02391

**Published:** 2018-10-30

**Authors:** Xiaopeng Jing, Huan Zhou, Xiaochun Min, Xing Zhang, Qing Yang, Shuaixian Du, Yirong Li, Fangyou Yu, Min Jia, Yu Zhan, Yi Zeng, Bo Yang, Yunjun Pan, Binghuai Lu, Rong Liu, Ji Zeng

**Affiliations:** ^1^Department of Clinical Laboratory, Wuhan Fourth Hospital, Puai Hospital, Tongji Medical College, Huazhong University of Science and Technology, Wuhan, China; ^2^Department of Pathophysiology, School of Basic Medicine, Key Laboratory of Ministry of Education of China for Neurological Disorders, Tongji Medical College, Huazhong University of Science and Technology, Wuhan, China; ^3^Department of Clinical Laboratory, First Affiliated Hospital, College of Medicine, Zhejiang University, Hangzhou, China; ^4^Department of Clinical Laboratory, Union Hospital, Huazhong University of Science and Technology, Wuhan, China; ^5^Department of Clinical Laboratory, Zhongnan Hospital, Wuhan University, Wuhan, China; ^6^Department of Clinical Laboratory, Shanghai Pulmonary Hospital, Tongji University School of Medicine, Shanghai, China; ^7^Department of Clinical Laboratory, Wuhan First Hospital, Wuhan, China; ^8^Department of Clinical Laboratory, The Central Hospital of Wuhan, Wuhan, China; ^9^Department of Clinical Laboratory, Renmin Hospital, Hubei University of Medicine, Shiyan, China; ^10^Department of Laboratory Medicine, Civil Aviation General Hospital, Peking University Civil Aviation School of Clinical Medicine, Beijing, China

**Keywords:** carbapenemase, modified carbapenem inactivation method, gram-negative bacilli, *Enterobacteriaceae*, simplified carbapenem inactivation method

## Abstract

This study reports the simplified carbapenem inactivation method (sCIM) to detect carbapenemase-producing gram-negative bacilli in a simple and accurate manner. This method is based on the modified carbapenem inactivation method (mCIM) with the improvement of experimental procedures. Instead of incubating the antibiotic disk in the organism culture media, the organism to be tested was smeared directly onto the antibiotic disk in the sCIM. For evaluating the sensitivity and specificity of the method, a total of 196 *Enterobacteriaceae*, 73 *Acinetobacter baumannii*, and 158 *Pseudomonas aeruginosa* isolates were collected. Polymerase chain reaction (PCR) was used to detect the carbapenemase genes. Phenotypic evaluations were performed using both the sCIM and the mCIM. PCR results showed that, of the 196 *Enterobacteriaceae* strains, 147 expressed the carbapenemase genes *bla_KPC−2_* (58.5%), *bla_IMP−4_* (21.8%), *bla_IMP−2_* (2.0%), *bla_VIM−1_* (6.1%), *bla_NDM−1_* (10.2%), and *bla_OXA−48_* (1.4%). sCIM results had high concordance with PCR results (99.5%) and mCIM results (100%) with the exception of one *Klebsiella pneumoniae* strain, which had an minimal inhibitory concentration (MIC) for imipenem of 0.25 mg/L. PCR demonstrated that 53 of the 73 *A. baumannii* isolates expressed the carbapenemase genes *bla_OXA−23_* (98.1%) and *bla_VIM−2_* (1.8%). sCIM and PCR results corresponded but all *A. baumannii* isolates were carbapenemase negative by the mCIM. PCR demonstrated that 25 of the 158 *P. aeruginosa* isolates expressed carbapenemase genes *bla_VIM−1_* (52%)*_,_ bla_VIM−2_* (8%)*_,_ bla_VIM−4_* (36%), and *bla_IMP−4_* (4%). sCIM results had high concordance with PCR results (100%) and the mCIM results (99.4%) with the exception of one *P. aeruginosa* isolate that expressed the *bla_VIM−4_* gene. The sCIM offers specificity and sensitivity comparable to PCR but has the advantage of being more user-friendly. This method is suitable for routine use in most clinical microbiology laboratories for the detection of carbapenemase-producing gram-negative bacilli.

## Introduction

Carbapenemases can be divided based on their molecular characteristics into class A, B, or D using the Ambler classification system. Class A and D carbapenemases require serine at their active sites, whereas class B carbapenemases, also called metallo-β-lactamases (MBLs), require zinc for β-lactam hydrolysis (Jean et al., 2015; Bonomo, 2017; Khan et al., 2017). The most common class A carbapenemases are KPC enzymes, while notable transmissible class B carbapenemase include IMP, VIM, and NDM enzymes. Common class D carbapenemases include OXA-23-like, OXA-24-like, OXA-48-like, and OXA-58-like enzymes (Jean et al., 2015).

The prevalence of carbapenemases in gram-negative bacilli, especially carbapenemase-producing *Enterobacteriaceae* (CPE), *Acinetobacter baumannii*, and *Pseudomonas aeruginosa*, has increased markedly in the past 10 years (Jean et al., 2015; Logan and Weinstein, 2017). Genes encoding carbapenemases are often located on plasmids, facilitating the spread of carbapenem resistance between different bacteria (Jean et al., 2015). Carbapenem-resistant strains have caused difficulties in the clinical treatment and prevention of nosocomial infections.

The convenient and accurate detection of carbapenemases are of great clinical importance. CLSI (2010) introduced the modified Hodge test for carbapenemase detection, but this method can only be used for the accurate detection of KPC-type carbapenemases in *Enterobacteriaceae*. CLSI (2012) recommended the Carba NP test method for the detection of carbapenemases in gram-negative bacilli; however, the preparation of the reagents required for this test is complicated and the solutions cannot be stored for extended periods, limiting its clinical application. van der Zwaluw et al. (2015) designed a new detection method, carbapenem inactivation method (CIM), which is easy to operate and highly sensitive in the detection of carbapenemases. In 2017, based on the CIM method, CLSI recommended the modified carbapenem inactivation method (mCIM). This method is effective at detecting a variety of carbapenemases (CLSI, 2017; Pierce et al., 2017). However, it is a relatively complex method and can only be used to detect carbapenemases in *Enterobacteriaceae* and *P. aeruginosa* (CLSI, 2018). In the present study, based on the mCIM, we designed a simplified carbapenem inactivation method (sCIM) for simple and accurate detection of carbapenemases in gram-negative bacilli and compared it with polymerase chain reaction (PCR) and mCIM methods.

## Materials and Methods

### Bacteria

To validate the sCIM, we collected 194 *Enterobacteriaceae*, 73 *A. baumannii*, and 158 *P. aeruginosa* clinical isolates from eight hospitals in China during 2017, and two OXA-48-producing *Klebsiella pneumoniae* and *Escherichia coli* from clinically conserved strains (Yu et al., 2017). Microorganisms were identified using the Microflex LT system (Bruker Daltonik GmbH, Bremen, Germany) and minimal inhibitory concentration (MIC) of imipenem, meropenem, and ertapenem were determined using the broth microdilution method. The 194 *Enterobacteriaceae* clinical isolates included 104 strains of *K. pneumoniae*, 72 strains of *E. coli*, and 18 strains of *Enterobacter cloacae*, in which 146 strains were resistant to imipenem and meropenem and five strains were resistant to ertapenem but susceptible to imipenem and meropenem. The two OXA-48-producing *K. pneumoniae* and *E. coli* were intermediate to imipenem, meropenem, and ertapenem. Fifty-three strains of *A. baumannii* and 149 strains of *P. aeruginosa* were resistant to imipenem and meropenem.

### Modified Carbapenem Inactivation Method

In the mCIM, 1 μL loopfuls of *Enterobacteriaceae* or 10 μL loopfuls of *P. aeruginosa* or *A. baumannii* from blood agar plates was emulsified in 2 mL trypticase soy broth (TSB). A meropenem disk was then immersed in the suspension and incubated for a minimum of 4 h at 35°C. A 0.5 McFarland suspension of *E. coli* ATCC 25922 was prepared in saline using the direct colony suspension method. A Mueller–Hinton agar (MHA) plate was inoculated with *E. coli* ATCC 25922 using the routine disk diffusion procedure. The meropenem disk was removed from the TSB and placed on an MHA plate previously inoculated with the *E. coli* ATCC 25922 indicator strain. Plates were incubated at 35°C in ambient air for 18−24 h. An inhibition zone diameter of 6–15 mm or colonies within a 16–18 mm zone was considered to be a positive result, and a zone of inhibition ≥19 mm was considered to be a negative result (CLSI, 2017; Pierce et al., 2017; CLSI, 2018).

### Simplified Carbapenem Inactivation Method

**FIGURE 1:**
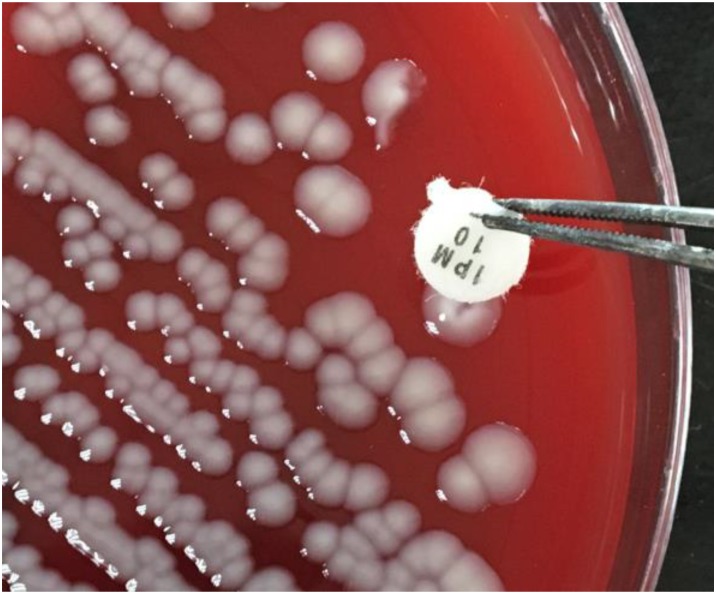
The sCIM testing procedure. Bacteria to be tested were grown overnight on a blood agar plate. One to three overnight colonies were smeared onto an imipenem disk and the disk was then placed on the testing plate.

**FIGURE 2:**
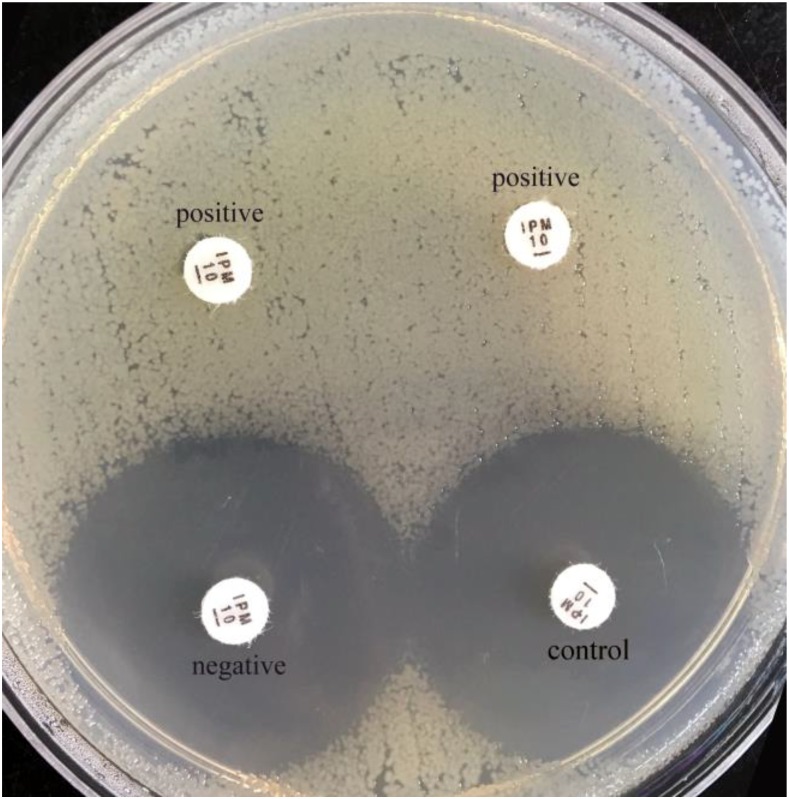
Results of sCIM testing of gram-negative bacilli. The zones of inhibition of the negative isolate and the control were similar, whereas the zones of inhibition of the positive isolates (the left is a *K. pneumonia* producing KPC-2; the right is an *E. coli* producing NDM-1) were 6 mm.

**FIGURE 3:**
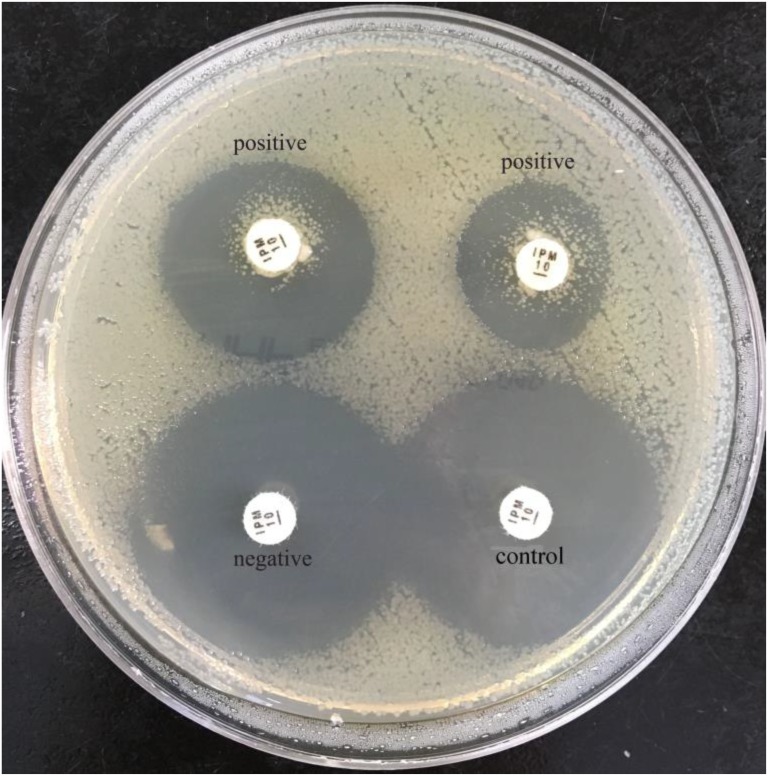
Results of sCIM testing of gram-negative bacilli. Positive isolates (the left is a *P. aeruginosa* producing VIM-4; the right is an *A. baumannii* producing OXA-23) demonstrate satellite growth within the zone of inhibition around the disk.

The sCIM is based on the mCIM with improvement of experimental procedures. Instead of incubating the antibiotic disk in the organism culture media for 4 h as in the mCIM, the organism to be tested was smeared directly onto an antibiotic disk in the sCIM. To perform the sCIM, for *Enterobacteriaceae*, a 0.5 McFarland standard suspension (using the direct colony suspension method) of *E. coli* ATCC 25922 was inoculated onto the MHA plate, following the routine disk diffusion procedure; for *A. baumannii* and *P. aeruginosa*, a 0.5 McFarland standard suspension (using direct colony suspension method) of *E. coli* ATCC 25922 was diluted 1:10 in saline and inoculated onto the MHA plate, following the routine disk diffusion procedure. Plates were allowed to dry for 3–10 min. Then, 1–3 overnight colonies of the test organisms grown on blood agar were smeared onto an imipenem disk (10 μg; Oxoid, Hampshire, United Kingdom) (Figure 1) to allow one side of the disk was evenly coated with the test bacteria; immediately afterward, the side of the disk having bacteria was placed on the MHA plate previously inoculated with *E. coli* ATCC 25922. An imipenem disk placed on an MHA plate was used as the control. All plates were incubated at 35°C for 16–18 h in ambient air. Bacterial strains that produced carbapenemase can hydrolyze imipenem; hence. the susceptible indicator strain grew unchecked. In contrast, the zone of inhibition around the disk shows a diameter of 6–20 mm (Figure 2), or the satellite growth of colonies of *E. coli* ATCC 25922 around the disk with a zone diameter ≤22 mm (Figure 3), indicating that the isolate was capable of producing carbapenemase; a zone of inhibition ≥26 mm was considered to be a negative result; a zone of inhibition of 23–25 mm was considered to be a carbapenemase indeterminate result.

### PCR Detection of Carbapenemase Genes

To perform PCR, primers were designed to detect the *bla*_KPC_, *bla*_IMP_, *bla*_VIM_, *bla*_NDM_, *bla*_OXA−48−like_, and *bla*_OXA−23−like_ genes (Table 1). PCR was performed according to previously described procedures (Yigit et al., 2001; Woodford et al., 2006; Doyle et al., 2012). Briefly, 25 μL of PCR Master Mix (CWBio, Beijing, China) was mixed with 4 μL of forward and reverse primers and water to a final volume of 45 μL. Then, 5 μL of sample lysate from each test isolate was added to the mix. For *Enterobacteriaceae* and *P. aeruginosa*, the PCR program consisted of an initial denaturation step at 94°C for 5 min, followed by 35 cycles of denaturation at 94°C for 10 s, annealing at 56°C for 30 s, elongation at 72°C for 90 s, and a final extension at 72°C for 2 min. For *A. baumannii*, the PCR program consisted of an initial denaturation step at 94°C for 5 min, followed by 35 cycles of denaturation at 94°C for 25 s, annealing at 52°C for 40 s, elongation at 72°C for 50s, and a final extension at 72°C for 6 min. PCR products were selected for sequencing and sequences were aligned using the BLAST software tool^1^.

**Table 1:** Primers for the detection of carbapenemase-producing *Enterobacteriaceae.*

Carbapenemase gene	Primer sequences^a^	Amplicon size (bp)	Reference
*bla*_KPC_	5′-TGTCACTGTATCGCCGTC-3′	1010	Khan et al., 2017
	5′-CTCAGTGCTCTACAGAAAACC-3′		
*bla*_IMP_	5′-GAAGGCGTTTATGTTCATAC-3′	587	Jean et al., 2015
	5′-GTACGTTTCAAGAGTGATGC-3′		
*bla*_VIM_	5′-GTTTGGTCGCATATCGCAAC-3′	389	Jean et al., 2015
	5′-AATGCGCAGCACCAGGATAG-3′		
*bla*_NDM_	5′-GCAGCTTGTCGGCCATGCGGGC-3′	782	Jean et al., 2015
	5′-GGTCGCGAAGCTGAGCACCGCAT-3′		
*bla*_OXA−48−like_	5′-GCGTGGTTAAGGATGAACAC-3′	438	Jean et al., 2015
	5′-CATCAAGTTCAACCCAACCG-3′		
*bla*_OXA−23−like_	5′-GATCGGATTGGAGAACCAGA-3′	501	Li et al., 2012
	5′-ATTTCTGACCGCATTTCCAT-3′		

*^a^The first and second primers for each gene are forward and reverse primers, respectively.*

### sCIM Detection on Positive Blood Cultures

To further confirm the sensitivity of the sCIM in clinical detection, in August 2018, 47 gram-negative bacilli of positive blood cultures collected from three hospitals were directly tested with the sCIM and antimicrobial susceptibility tests at the same time. The positive rate of carbapenemases-producing strains was analyzed to assess if the method may shorten the turnaround time (TAT).

## Results

### Sensitivity and Specificity of sCIM

Of the 196 *Enterobacteriaceae* tested, 148 were shown to produce carbapenemase by the sCIM test, whereas 147 were shown to carry a carbapenemase-encoding gene by PCR. Both the OXA-48-producing *K. pneumoniae* and *E. coli* were carbapenemase positive by the sCIM and their MICs for imipenem were 2 mg/L. One *K. pneumoniae* isolate with an MIC for imipenem of 0.25 mg/L harbored CTX-M-15 and was found to be carbapenemase positive by the sCIM but carbapenemase negative by PCR. One *K. pneumoniae* isolate with an MIC for imipenem of 16 mg/L was found to be carbapenemase negative by both PCR and the sCIM, suggesting that this isolate may be a carbapenemase negative CRE (Table 2).

**Table 2:** Laboratory detection of 196 *Enterobacteriaceae* isolates.

*Enterobacteriaceae* (n)	PCR (n)	MIC of imipenem (mg/L)			MIC of meropenem (mg/L)			MIC of ertapenem (mg/L)			sCIM		mCIM	
		≥4	2	≤1	≥4	2	≤1	≥2	1	≤0.5	+	−	+	−
*K. pneumoniae* (105)	KPC-2 (54)	54	0	0	54	0	0	54	0	0	54	0	54	0
	IMP-4 (19)	19	0	0	19	0	0	19	0	0	19	0	19	0
	IMP-2 (1)	1	0	0	1	0	0	1	0	0	1	0	1	0
	VIM-1 (6)	6	0	0	6	0	0	6	0	0	6	0	6	0
	NDM-1 (3)	3	0	0	3	0	0	3	0	0	3	0	3	0
	OXA-48(1)	0	1	0	0	1	0	0	1	0	1	0	1	0
	None (21)	1	0	20	1	0	20	4	0	17	1	20	1	20
*E. coli* (73)	KPC-2 (31)	31	0	0	31	0	0	31	0	0	31	0	31	0
	IMP-4 (9)	9	0	0	9	0	0	9	0	0	9	0	9	0
	IMP-2 (2)	2	0	0	2	0	0	2	0	0	2	0	2	0
	NDM-1 (5)	5	0	0	5	0	0	5	0	0	5	0	5	0
	OXA-48(1)	0	1	0	0	1	0	0	1	0	1	0	1	0
	None (25)	0	0	25	0	0	25	2	0	23	0	25	0	25
*E. cloacae* (18)	KPC-2 (1)	1	0	0	1	0	0	1	0	0	1	0	1	0
	IMP-4 (4)	4	0	0	4	0	0	4	0	0	4	0	4	0
	VIM-1 (3)	3	0	0	3	0	0	3	0	0	3	0	3	0
	NDM-1 (7)	7	0	0	7	0	0	7	0	0	7	0	7	0
	None (3)	0	0	3	0	0	3	0	0	3	0	3	0	3

*+, positive; −, negative; sCIM, simplified carbapenem inactivation method; mCIM, modified carbapenem inactivation method.*

Of the 73 *A. baumannii* strains, 53 were resistant to imipenem. These 53 strains were all found to be carbapenemase positive by the sCIM. Fifty-two strains were found to carry the *bla_OXA−23_* gene and one strain carry the *bla_VIM−2_* gene by PCR. Twenty imipenem-susceptible *A. baumannii* strains were negative by both the sCIM and PCR. The concordance rate of the sCIM and PCR for *A. baumannii* was 100% (Table 3).

**Table 3:** Laboratory detection of carbapenemase-producing *A. baumannii* and *P. aeruginosa*.

Species (n)	PCR (n)	MIC of imipenem (mg/L)		MIC of meropenem(mg/L)		sCIM		mCIM	
		≥8	≤2	≥8	≤2	+	−	+	−
*P.aeruginosa* (158)	IMP-4 (1)	1	0	1	0	1	0	1	0
	VIM-1 (13)	13	0	13	0	13	0	13	0
	VIM-2 (2)	2	0	2	0	2	0	2	0
	VIM-4 (9)	9	0	9	0	9	0	8	1
	None (133)	124	9	124	9	0	133	0	133
*A.baumannii* (73)	OXA-23 (52)	52	0	52	0	52	0	0	52
	VIM-2 (1)	1	0	1	0	1	0	0	1
	None (20)	0	20	0	20	0	20	0	20

*+, positive; −, negative; sCIM, simplified carbapenem inactivation method; mCIM, modified carbapenem inactivation method.*

Of the 158 *P. aeruginosa* strains tested, 149 were resistant to imipenem and 25 were found to be carbapenemase positive by the sCIM. The concordance rate of the sCIM and PCR for *P. aeruginosa* was 100% (Table 3).

In our experiment, the zones of inhibition of strains found to be carbapenemase negative by the sCIM ranged in size from 28 to 32 mm. In contrast, the zones of inhibition of strains found to be carbapenemase positive by the sCIM were 6 mm for *Enterobacteriaceae* isolates, 15 ± 4 mm for *A. baumannii* isolates, and 7 ± 3 mm for *P. aeruginosa* isolates (Figures 2, 3 Table 4). Many small colonies were observed in the zones ≥12 mm around the imipenem disks, demonstrating the “satellite phenomenon” (Figure 3).

**Table 4:** Comparison of sCIM and mCIM results for selected strains.

Strain (n)	Type of carbapenemase	sCIM zone diameter (mm)	mCIM zone diameter (mm)
*Enterobacteriaceae* (147)	KPC-2, IMP-4, IMP-2, VIM-1, NDM-1, OXA-48	6	6
*P. aeruginosa* (23)	VIM-1, VIM-2, VIM-4	6	6−12
*P. aeruginosa* (1)	VIM-4	21^∗^	22
*P. aeruginosa* (1)	IMP-4	6	6
*A. baumannii* (52)	OXA-23	6−20^∗^	21−24
*A. baumannii* (1)	VIM-2	18^∗^	24

*sCIM, simplified carbapenem inactivation method; mCIM, modified carbapenem inactivation method. ^∗^Satellite growth observed around the disk within the zone of inhibition.*

### Carbapenemase Enzymes Produced by Tested Strains

In this study, 225 isolates were found to be positive for carbapenemase-encoding genes by PCR. The carbapenemases identified in *Enterobacteriaceae* isolates were KPC-2 (58.5%), IMP-4 (21.8%), IMP-2 (2.0%), VIM-1 (6.1%), NDM-1 (10.2%), and OXA-48 (1.4%) (Table 2). The carbapenemases identified in *A. baumannii* isolates were OXA-23 (98.1%) and VIM-2 (1.9%). The carbapenemases identified in *P. aeruginosa* were VIM-1 (52%), VIM-2 (8%), VIM-4 (36%), and IMP-4 (4%) (Table 3).

### The Sensitivity and Specificity of the sCIM Is Comparable to the mCIM

The results of tests of 196 *Enterobacteriaceae* showed that the concordance rate of the sCIM and the mCIM was 100%, including one false positive. Of the 158 *P. aeruginosa* isolates, 25 were found to be carbapenemase positive by the sCIM and 24 were found to be carbapenemase positive by the mCIM. The results were inconsistent for one VIM-4-producing isolate. Of the 73 *A. baumannii* isolates, 53 were found to be carbapenemase positive by the sCIM but all were found to be carbapenemase negative by the mCIM (Tables 2, 3). These results showed that the sCIM and the mCIM had similar rates of detection for CPE and *P. aeruginosa* and that the sCIM may be superior to the mCIM for the detection of carbapenemase-producing *A. baumannii*.

### sCIM Detection on Positive Blood Cultures

A total of 47 gram-negative bacteria isolated from blood cultures in three hospitals were tested by the sCIM. Eight *K. pneumoniae* producing KPC-2 and one *A. baumannii* producing OXA-23 isolates were positive, with a positive rate of 19.1% (9/47). No false-negative strains were found. These data indicate that the sCIM can directly detect blood culture positive strains and report the enzyme-producing strains 1 day earlier than the routine antimicrobial susceptibility test.

## Discussion

The detection principles of the sCIM and the mCIM are similar, based on the fact that carbapenemases can hydrolyze carbapenem (Queenan and Bush, 2007; Jean et al., 2015; van der Zwaluw et al., 2015; Bonomo, 2017; Pierce et al., 2017). However, the strategies of the two methods to hydrolyze carbapenem are different. In the mCIM, the antibiotic disk is put into the TSB containing the test organisms for approximately 4 h, whereas in the sCIM, the test organisms are directly smeared onto the antibiotic disk. The mCIM requires TSB and MHA plates, while the sCIM only requires MHA plates. Thus, the operation in the sCIM experiment has fewer steps and is more convenient than the mCIM. When carbapenemase-producing bacteria is smeared on imipenem disks, the enzyme spreads and hydrolyzes the antibiotics on the paper, leading to a reduction in the size of the zone of inhibition.

Another difference of the sCIM compared with the mCIM is that the imipenem disk is selected. In general, the different types of carbapenemases have different hydrolysis rates for different carbapenem (Jean et al., 2015). For most carbapenemases, the *K*m values for imipenem is much larger than that for meropenem (Queenan and Bush, 2007). Therefore, we compared the efficiency of different carbapenem disks in detecting carbapenemases in the current study. We found that the diameter of the zone of inhibition was 6 mm for all the testing CPEs when using the imipenem disk. When using the meropenem disk, the diameter of the zone of inhibition varied considerably in different CPEs, indicating that imipenem was more rapidly hydrolyzed by carbapenemases compared to meropenem. For an easier interpretation of the experimental results, we chose an imipenem disk for the sCIM test. Because a few of ESBLs or AmpC produced by *Enterobacteriaceae* might hydrolyze imipenem with low activity (Carvalhaes et al., 2010; Bialek-Davenet et al., 2017; van Boxtel et al., 2017), it may result in few false positives in the sCIM test. In our study, we discovered a false positive result in a *K. pneumoniae* strain producing CTX-15. but the MIC of imipenem was less than 2 mg/L for this isolate. However, no false-positive isolate was discovered from those with a MIC for imipenem ≥2 mg/L. Therefore, the sCIM may be suitable for the determination of carbapenem-nonsusceptible strains, while the carbapenem-susceptible strains may have few false positives.

The concentration of bacteria in the disk diffusion method can affect the diameter of the zone of inhibition. At the same concentration of the drug, the lower the concentration of the tested bacterial used, the larger the diameter of the zone of inhibition that will be obtained. Correspondingly, when the concentration of bacteria is lower, the change of the zone of inhibition is also greater upon the variation of antibiotics concentration. In general, the hydrolysis rate of carbapenem by carbapenemase-producing non-fermenting bacteria is weaker than that of CPE. Therefore, reducing the concentration of *E. coli* ATCC 25922 can increase the sensitivity of the sCIM in detecting non-fermenting bacteria. For initial experiments with non-fermenting bacteria, the concentration of *E. coli* ATCC 25922 was adjusted from the 0.5 McFarland standard suspension by 5-fold and 10-fold dilutions. We found that the results obtained using these two dilutions were similar for *A. baumannii*. However, only 24 strains of *P. aeruginosa* were carbapenemase positive at the fivefold dilution, whereas 25 strains were carbapenemase positive at the 10-fold dilution. Therefore, the concordance rate with PCR was higher at the 10-fold dilution than the 5-fold dilution. Based on these results, we selected a 10-fold dilution of the 0.5 McFarland standard suspension of *E. coli* ATCC25922 for experiments with *P. aeruginosa* and *Acinetobacter* spp.

PCR results showed that KPC, VIM, NDM, IMP, and OXA-type enzymes were produced by *Enterobacteriaceae*, *A. baumannii*, and *P. aeruginosa*, and the sCIM can be used for sensitive (100%) and specific (99.6%) detection of carbapenemase-producing gram-negative bacilli. Only one of the 226 sCIM positive isolates, a CTX-15-producing *K. pneumoniae* strain, caused a false positive in sCIM tests. Pierce et al. (2017) reported that TEM-1 and TEM-52 enzymes might cause false positives in mCIM tests, too. CLSI (2018) recommended that the mCIM be used to detect carbapenemases in *Enterobacteriaceae* and *P. aeruginosa*. We found that only one isolate carrying the gene encoding VIM-4 was carbapenemase negative by the mCIM in our tests. Therefore, we concluded that the sensitivity of the sCIM for detecting CPE and *P. aeruginosa* was similar to that of the mCIM.

The carbapenemases identified in *Enterobacteriaceae* isolates were mainly KPC-2 and IMP-4. Because mainland China is not an OXA-48-endemic area, no OXA-48 positive strains were found in our collected isolates. These results were similar to the distribution of enzymes reported in mainland China by Zhang et al. (2017). To confirm the capability of the sCIM to detect OXA-48 carbapenemase, we collected two clinically conserved OXA-48-producing strains. Both strains were positive by the sCIM and the mCIM. However, we still cannot completely evaluate the capability of the sCIM to detect OXA-48 because of the small number of tests, and more investigations are needed to further confirm the reliability of the sCIM in detecting OXA-48-expressing isolates.

Our experimental data showed that the majority of CRE was CPE (99.3%), and only one *K. pneumoniae* isolate with an MIC for imipenem of 16 mg/L was not producing carbapenemase. This isolate was resistant to beta-lactams and susceptible to aminoglycosides and fluoroquinolones. The ratio of carbapenemase-producing isolates was 16.8% in carbapenem-resistant *P. aeruginosa*, and was slightly higher than the data reported by Yin et al. (2018). Carbapenemase-producing isolates are only a small part of carbapenem-resistant *P. aeruginosa*, and the loss or alteration of OprD is thought to be the most prevalent mechanism for carbapenem resistance in *P. aeruginosa* (Li et al., 2012; Yin et al., 2018). Among the 53 carbapenem-resistant *A. baumannii*, 52 produced OXA-23 type enzyme, and one produced VIM-2 type enzyme. The resistance mechanism of carbapenem-resistant *A. baumannii* is mainly related to carbapenemase (Jean et al., 2015).

Compared to other carbapenemase detection methods, the sCIM has several obvious advantages. First, the sCIM does not require special equipment or reagents, hence is less costly. Second, the sCIM is easy to perform using the conventional paper diffusion method and is not complicated. Third, the results can be easily assessed. Finally, the sCIM has a wide range of detection and can be used to detect CPE*, Acinetobacter* spp., and *P. aeruginosa*. The sCIM is suitable for routine use in most clinical microbiology laboratories to detect carbapenemase-producing bacteria and can contribute to the reduction of carbapenemase-producing gram-negative bacilli in hospitals.

## Author Contributions

XJ, XM, XZ, QY, SD, YL, MJ, YiZ, YuZ, BY, YP, FY, and BL isolated the bacteria and performed the laboratory measurements. JZ, XJ, and HZ made substantial contributions to conception and design. JZ and RL wrote and revised the manuscript. JZ drafted the manuscript. All authors read and approved the final manuscript.

## Conflict of Interest Statement

The authors declare that the research was conducted in the absence of any commercial or financial relationships that could be construed as a potential conflict of interest.

## Footnotes

1 https://blast.ncbi.nlm.nih.gov/Blast.cgi
